# Convergent validity of K-SADS-PL by comparison with CBCL in a Portuguese speaking outpatient population

**DOI:** 10.1186/1471-244X-10-83

**Published:** 2010-10-19

**Authors:** Heloisa HA Brasil, Isabel A Bordin

**Affiliations:** 1 Child and Adolescent Psychiatry Division, Institute of Psychiatry, Universidade Federal do Rio de Janeiro, Rua Gomes Carneiro 64/301 - Ipanema, CEP: 22071-110, Rio de Janeiro, RJ, Brazil; 2 Social Psychiatry Division, Department of Psychiatry, Federal University of São Paulo, Rua Borges Lagoa 570/cj 51, 04038-030, São Paulo, SP, Brazil

## Abstract

**Background:**

Different diagnostic interviews in child and adolescent psychiatry have been developed in English but valid translations of instruments to other languages are still scarce especially in developing countries, limiting the comparison of child mental health data across different cultures. The present study aims to examine the convergent validity of the Brazilian version of the Schedule for Affective Disorders and Schizophrenia for School-Age Children/Present and Lifetime Version (K-SADS-PL) by comparison with the Child Behavior Checklist (CBCL), a parental screening measure for child/adolescent emotional/behavior problems.

**Methods:**

An experienced child psychiatrist blind to CBCL results applied the K-SADS-PL to a consecutive sample of 78 children (6-14 years) referred to a public child mental health outpatient clinic (response rate = 75%). Three K-SADS-PL parameters were considered regarding current disorders: parent screen interview rates, clinician summary screen interview rates, and final DSM-IV diagnoses. Subjects were classified according to the presence/absence of any affective/anxiety disorder, any disruptive disorder, and any psychiatric disorder based on K-SADS-PL results. All subjects obtained T-scores on CBCL scales (internalizing, externalizing, total problems).

**Results:**

Significant differences in CBCL mean T-scores were observed between disordered and non-disordered children. Compared to children who screened negative, children positive for any affective/anxiety disorder, any disruptive disorder, and any psychiatric disorder had a higher internalizing, externalizing and total problem T-score mean, respectively. Highly significant differences in T-score means were also found when examining final diagnoses, except for any affective/anxiety disorder.

**Conclusions:**

Evidence of convergent validity was found when comparing K-SADS-PL results with CBCL data.

## Background

Reliable epidemiological data on the prevalence of psychiatric disorders among children and adolescents, risk and protective factors, comorbidity, and service utilization is highly relevant for service planning and health policy decisions in any country [1-4]. However, there is need for greater attention to the development of epidemiological assessment tools to suit local conditions [5]. Research tools and methods should not be imported from one country to another without careful analysis of the influence and effect of cultural factors on their reliability and validity. In addition, scientific tools need to be further developed to allow valid international comparisons that will help in understanding the commonalities and differences in the nature of mental disorders and their management across different cultures [6].

Regarding child psychopathology research, it is important for every country to have screening and diagnostic instruments that show convergent validity. In order to reduce costs of large epidemiological studies, child mental health evaluation is usually performed in two consecutive phases. First, a screening instrument is applied to the entire sample to identify suspected cases, and second, a diagnostic instrument is applied to all positive children (a smaller number) and to a representative sample of negative children (a bigger number). This strategy favors the study feasibility, but if the screening and the diagnostic instruments do not have convergent validity, the quality of data collected may be compromised.

A literature review based on PubMed (Publisher's MEDLINE), SciELO (Scientific Electronic Library Online) and LILACS (Latin American and Caribbean Health Sciences Literature) showed that valid diagnostic instruments in child psychiatry are still scarce in Brazil. The need of having a valid diagnostic instrument useful in clinical and epidemiological research motivated the development of the Brazilian version of the Schedule for Affective Disorders and Schizophrenia for School-Age Children - Present and Lifetime Version (K-SADS-PL), and the study of its convergent validity.

The K-SADS-PL [7] is a semi-structured diagnostic interview designed by Kaufman et al. in 1996 to assess current and past episodes of psychopathology in children and adolescents. The Brazilian version of the K-SADS-PL (in Portuguese) was developed by Brasil and Bordin from the original English version with the author's permission. Its development occurred under rigorous methodological requirements regarding translation, back-translation, cultural adaptation and study of psychometric properties [8].

This is the first study conducted in Brazil to examine the convergent validity of a psychiatric diagnostic interview for children and adolescents (Brazilian version of K-SADS-PL) by comparison with a parental screening instrument for child and adolescent emotional and behavioral problems that is internationally recognized by its quality and usefulness (CBCL). Because children with high values on behavior problem scales have a high probability of being classified as a case by a psychiatrist [9], we hypothesize that CBCL scores will be correlated to K-SADS-PL results. When seeing how closely our measure of child psychopathology (K-SADS-PL) is related to other measures of the same construct to which it should be related (CBCL) consists in the assessment of convergent validity [10].

The aim of this study is to examine the convergent validity of the Brazilian version of K-SADS-PL by comparison with a parental screening measure for child and adolescent emotional/behavior problems (CBCL) that is extensively used internationally and validated in Brazil.

## Methods

### Participants

The present study was conducted with a consecutive sample of children (n = 78) scheduled for first appointment at the child mental health outpatient clinic of the Federal University of Rio de Janeiro. That university outpatient clinic is a public service free of charge that typically assists children from low-income families. Because sources of referral include health professionals, schools, social services, and parents themselves, the group of children scheduled for first appointment is heterogeneous in terms of psychopathology, including children without disorders and clinical cases of different severity levels.

Inclusion criteria encompassed children of both genders aged 6-14 years with a parent/caregiver currently living with them that could provide a history about the child's symptoms. The following exclusion criteria were applied: (1) child in bad physical health condition in urgent need of care (e.g. severe anorexia); (2) autistic, mentally retarded, psychotic or organic brain syndrome; and (3) parent/caregiver not able to give coherent verbal information (e.g. mental retardation, active psychosis). Participants (n = 78) represented 75% of the total number of eligible children scheduled for first appointment at the child mental health outpatient clinic of the Federal University of Rio de Janeiro in 28 consecutive weeks (2001).

### Instruments

#### The Schedule for Affective Disorders and Schizophrenia for School-Age Children/Present and Lifetime Version (K-SADS-PL)

The K-SADS-PL is a semi-structured psychiatric interview that ascertains both lifetime and current diagnostic status [11] based on DSM-IV criteria [12]. A current episode of disorder refers to the period of maximum severity within the episode (symptom free period not greater than two months). K-SADS-PL includes three components: introductory interview (demographic, health, and other background information), screen interview (82 symptoms related to 20 diagnostic areas), and five diagnostic supplements: (1) affective disorders (major depression, dysthymia, mania, hypomania); (2) psychotic disorders; (3) anxiety disorders (social phobia, agoraphobia, specific phobia, obsessive-compulsive disorder, separation anxiety disorder, generalized anxiety disorder, panic disorder, posttraumatic stress disorder); (4) disruptive behavioral disorders (attention deficit hyperactivity disorder/ADHD, conduct disorder, oppositional defiant disorder); and (5) substance abuse, tic disorders, eating disorders, and elimination disorders (enuresis, encopresis).

The skip-out criteria in the screen interview specify which sections of the supplements, if any, should be completed. The skip-out criteria take into account the threshold of symptom severity from each of the 82 screening items for 20 diagnostic areas. Just one screening item from determined diagnostic area achieving the threshold indicates the need of further assessment with complementary items from the same diagnostic area that are included in the related supplement. When none of the 82 symptoms achieve the threshold, no supplement is applied, and we can consider absent the related 20 psychiatric diagnoses (major depression, dysthymia, mania, hypomania, psychotic disorders, social phobia, agoraphobia, specific phobia, obsessive-compulsive disorder, separation anxiety disorder, generalized anxiety disorder, panic disorder, posttraumatic stress disorder, ADHD, conduct disorder, oppositional defiant disorder, substance abuse, tic disorders, eating disorders, and elimination disorders). The administration technique involves first the clinical interview with the parent alone to obtain the parent screening interview score, and second the same interview with the child alone applied by the same clinician to obtain the child screening interview score. After interviewing parent and child, a summary rating is made by the clinician based on all sources of information available and the use of her/his clinical judgment (clinician's screening interview summary score).

As a semi-structured diagnostic interview to be used in child psychiatry clinical practice and child mental health research, it requires clinical experience and extensive training. Clinical skills on the part of interviewers depend on acquired knowledge about child development and psychopathology. Clinicians must be aware of the importance of using their best clinical judgment when integrating information from children and caregivers, and of taking into account familial and socio-cultural factors when interpreting informant answers. Additionally, substantial familiarity with the instrument content, skip-out rules, threshold and subthreshold definitions, and DSM-IV criteria are essential to the correct scoring of K-SADS-PL items.

The Brazilian version of K-SADS-PL was developed from the original English version^7 ^using recommended procedures for translation, back-translation and cultural adaptation [13-16]. Three Brazilian experienced professionals (two child psychiatrists and one psychologist) were responsible for the translation to Portuguese with special attention to different dimensions of equivalence including cultural adaptation. Extensive field-testing helped find adequate wording understandable by children and low-educated parents. A final version was submitted to back-translation by a North-American professional translator blind to the original version of K-SADS-PL. Once translation and back-translation were completed, validity of the instrument was examined within the new context as recommended by Streiner and Norman [10].

#### The Child Behavior Checklist (CBCL/4-18)

The CBCL/4-18 is a standardized parent-report questionnaire designed by Achenbach (1991) [17] to assess emotional and behavior problems and social competencies in children with good validity and reliability. The emotional/behavior problem section of CBCL/4-18 has 118 items, and provides scores for three broad-band scales: internalizing (sum of subscales withdrawn, somatic complains and anxious/depressed), externalizing (sum of subscales delinquent behavior and aggressive behavior) and total behavior problem. Initial findings from a validity study [18] showed high sensitivity of the Brazilian version of CBCL/4-18 (developed by Bordin from the original English version [17] with the author's permission) when compared with ICD-10 psychiatric diagnoses made by an experienced child psychiatrist blind to CBCL/4-18 results. In a random sample of low-income pediatric outpatients (n = 49, 4-12 years), CBCL/4-18 was applied to mothers by a trained lay interviewer due to their low educational level, and 80.4% of children with one or more ICD-10 psychiatric diagnosis were in the CBCL/4-18 borderline or clinical range for total behavior problems (T-score ≥ 60). Considering all children with ICD-10 psychiatric diagnosis, the Brazilian version of CBCL/4-18 correctly identified 100% of severe cases, 95% of moderate cases, and 75% of mild cases [18]. In the present study, the Brazilian version of CBCL/4-18 was applied to mothers/caregivers to obtain standardized parents' reports of children's current emotional/behavior problems. All scales' raw scores were transformed into T-scores, which were used as continuous variables in the analysis. Children with emotional/behavior problems were those with broad-band scale T-scores in the clinical range (T-score > 63, above the 90th percentile according to the American normative sample). CBCL/4-18 T-scores varying from 60 to 63 characterized borderline cases.

In the present study, CBCL/4-18 was applied to parents/caregivers (usually the mother) by a trained interviewer up to two weeks prior to K-SADS-PL interview (n = 78). Parents and children were individually interviewed by an experienced child psychiatrist that administered the K-SADS-PL blind to CBCL/4-18 results. All parents/caregivers who participated in the study gave written informed consent in accordance with the Research Ethics Committee of the Pan American Health Organization, Federal University of São Paulo, and Federal University of Rio de Janeiro. All children provided oral consent and assent to participate.

### Analysis

The convergent validity of the Brazilian Version of K-SADS-PL was examined by comparison with CBCL/4-18 broad-band scale results.

Three K-SADS-PL parameters were considered regarding current disorders: parent screen interview rates, clinician screen interview rates (clinical judgment taking into account parent and child information), and final DSM-IV diagnoses. Based on these parameters, subjects were classified according to the presence or absence of any affective/anxiety disorder, any disruptive disorder (not including ADHD), and any psychiatric disorder. Affective disorders included depressive disorders, dysthymia, mania, hypomania, and bipolar disorder. Anxiety disorders included social phobia, agoraphobia, specific phobias, separation anxiety disorder, generalized anxiety disorder, obsessive compulsive disorder, panic disorder, acute stress disorder, and posttraumatic stress disorder. Disruptive disorders included oppositional defiant disorder and conduct disorder. When examining the convergent validity of K-SADS-PL compared to CBCL/4-18, ADHD was excluded from the group of disruptive disorders since attention problems are not part of the CBCL/4-18 externalizing scale. Any psychiatric disorder included all disorders covered by the K-SADS-PL.

According to the three K-SADS-PL parameters mentioned above, children with any disorder and children with no disorders were compared regarding CBCL/4-18 total behavior problem scale's mean scores; children with any affective/anxiety disorder and children without affective/anxiety disorders were compared regarding CBCL/4-18 internalizing scale's mean scores; and children with any disruptive disorder and children without disruptive disorders were compared regarding CBCL/4-18 externalizing scale's mean scores.

## Results

Study participants included 26 girls (mean age 10.1 ± 3.0) and 52 boys (mean age 9.8 ± 2.6). From these 78 children referred to first appointment at the child mental health outpatient clinic of the Federal University of Rio de Janeiro, 64% were aged 6-11 years, and 36% were aged 12-14 years. In that sample, 74.4% of children achieved the K-SADS-PL threshold for at least one current psychiatric disorder with disruptive disorders and anxiety disorders being more frequent than affective disorders or eating disorders (table 1). From the total number of children with any psychiatric disorder (n = 58), 21 (36.2%) received a single K-SADS-PL final diagnosis, while 37 (63.8%) achieved the threshold for two or more final diagnoses. Only eight out of 20 children with no K-SADS-PL final diagnoses were also negative in all 20 diagnostic areas of the clinician's screening interview. However, even those eight children were not asymptomatic since sub-threshold scores were obtained in two to seven items from the clinician's screening interview.

**Table 1:** Positive diagnostic areas in the screen interview and final diagnoses (N = 78)

	K-SADS-PL screen interview					
			
	Parent information		Clinical judgment		K-SADS-PL final diagnoses	
K-SADS-PL diagnostic areas (for the screen interview) or DSM-IV						
psychiatric disorders (for final diagnoses)*	N	(%)	N	(%)	N	(%)
AFFECTIVE DISORDERS						
Depressive disorders	5	(6.4)	5	(6.4)	4	(5.1)
Major depression disorder	NA	NA	NA	NA	2	(2.6)
Dysthymia	NA	NA	NA	NA	1	(1.3)
Depressive disorder NOE	NA	NA	NA	NA	1	(1.3)
Mania	0	(0.0)	0	(0.0)	0	(0.0)
ANXIETY DISORDERS						
Social Phobia	10	(12.8)	13	(16.7)	9	(11.5)
Agoraphobia	0	(0.0)	0	(0.0)	0	(0.0)
Specific Phobia	25	(32.1)	27	(34.6)	13	(16.7)
Obsessive-compulsive disorder	9	(11.5)	10	(12.8)	9	(11.5)
Separation anxiety disorder	19	(24.4)	23	(29.5)	11	(14.1)
Generalized anxiety Disorder	11	(14.1)	10	(12.8)	4	(5.1)
Panic disorder	0	(0.0)	0	(0.0)	0	(0.0)
Posttraumatic stress disorder	4	(5.1)	6	(7.7)	2	(2.6)
DISRUPTIVE DISORDERS						
ADHD	38	(48.7)	37	(47.4)	24	(30.8)
Oppositional defiant disorder	32	(41.0)	32	(41.0)	18	(23.1)
Conduct disorder	20	(25.6)	22	(28.2)	10	(12.8)
PSYCHOTIC DISORDERS	0	(0.0)	0	(0.0)	0	(0.0)
OTHER DISORDERS						
Substance abuse	0	(0.0)	0	(0.0)	0	(0.0)
Alcohol abuse	0	(0.0)	0	(0.0)	0	(0.0)
Drug abuse	0	(0.0)	0	(0.0)	0	(0.0)
Tic disorders	4	(5.1)	4	(5.1)	3	(3.8)
Motor	NA	NA	NA	NA	1	(1.3)
Transient	NA	NA	NA	NA	1	(1.3)
Tourette	NA	NA	NA	NA	1	(1.3)
Eating disorders	1	(1.3)	1	(1.3)	0	(0.0)
Anorexia	1	(1.3)	1	(1.3)	0	(0.0)
Bulimia	0	(0.0)	0	(0.0)	0	(0.0)
Eliminating disorders	13	(16.7)	13	(16.7)	13	(16.7)
Enuresis	12	(15.4)	12	(15.4)	12	(15.4)
Encopresis	1	(1.3)	1	(1.3)	1	(1.3)

NA = Not applicable (not part of K-SADS-PL screen interview)

* Multiple diagnoses are possible

Table 1 shows that many children with positive diagnostic areas in the K-SADS-PL screen interview according to the clinician did not have these diagnoses confirmed by the same clinician when completing the K-SADS-PL related supplements. This is especially true for anxiety disorders and disruptive behavior disorders (including ADHD). For instance, the clinician considered 27 children positive for specific phobia in the screen interview, but only 13 had specific phobia confirmed as a final diagnosis. Also, the clinician considered 22 children positive for conduct disorder in the screen interview, but only 10 had conduct disorder confirmed as a final diagnosis (table 1).

When looking at CBCL/4-18 results, 78% of our sample scored in the clinical range for total behavior problems, and high levels of internalizing (68.0%) and externalizing (60.3%) problems were noted with 44.9% of children presenting both internalizing and externalizing problems (table 2).

**Table 2:** Child emotional/behavioral problems according to CBCL* broad-band scales (N = 78)

CBCL/4-18 broad-band scales	N	(%)
Total problems		
Clinical**	61	(78.2)
Borderline	7	(9.0)
Non-clinical	10	(12.8)
Internalizing problems^a^		
Clinical**	53	(68.0)
Borderline	10	(12.8)
Non-clinical	15	(19.2)
Externalizing problems^b^		
Clinical**	47	(60.3)
Borderline	11	(14.1)
Non-clinical	20	(25.6)
Internalizing and externalizing problems combined		
Both scales**	35	(44.9)
Internalizing only	18	(23.1)
Externalizing only	12	(15.4)
None	13	(16.6)

* CBCL/4-18

** T scores in the clinical range (> 63)

^a ^Sum of CBCL subscales I, II & III (withdrawal, anxiety/depression, somatic complaints)

^b ^Sum of CBCL subscales VII & VIII (delinquent behavior, aggressive Behavior)

The Brazilian version of K-SADS-PL showed evidence of convergent validity when compared to CBCL/4-18. The group of children with one or more positive diagnostic areas in the parent screen interview scored significantly higher on CBCL/4-18 total problem scale than subjects with negative parental screen results (mean T-scores: 70.7 vs. 64.6, p = .015). The same was noted for the group of children with one or more positive diagnostic areas in the clinician screen interview compared to subjects with negative clinician screen results (mean T-scores: 70.7 vs. 62.7, p = .005), and for children with one or more final DSM-IV diagnosis compared to subjects with no disorders (mean T-scores: 71.1 vs. 66.1, p = .018) (table 3). In addition, children positive in one or more disruptive diagnostic areas in the parent screen interview had a higher mean T-score at the CBCL/4-18 externalizing scale than children negative in these investigated areas according to the parent (72.7 vs. 60.9, p < .001). Higher mean externalizing T-scores were also observed in children positive in one or more disruptive diagnostic areas in the clinician screen interview compared to children negative in these investigated areas according to the clinician (72.5 vs. 60.5, p < .001). When considering K-SADS-PL final diagnoses, children with one or more disruptive disorders had a higher mean T-score at the CBCL/4-18 externalizing scale than subjects with no disruptive disorders (74.9 vs. 62.5, p < .001). Similarly, children with K-SADS-PL positive screen results in one or more of the affective and/or anxiety diagnostic areas scored higher on CBCL/4-18 internalizing scale than subjects negative in these investigated areas (parent: 70.0 vs. 62.2, p < .001; clinician: 69.3 vs. 62.8, p = .004). However, when considering K-SADS-PL final diagnoses, the difference in means of CBCL/4-18 internalizing T-scores between children with one or more affective and/or anxiety disorders and subjects without any of these disorders only reached significance at a marginal level (p = .057) (table 3).

**Table 3:** Convergent validity of the Brazilian version of K-SADS-PL and CBCL/4-18 (N = 78)

	CBCL/4-18 broad-band scales											
	
	Total problems				Internalizing^a^				Externalizing^b^			
			
K-SADS-PL diagnostic areas (for the screen interview) or DSM-IV psychiatric disorders (for final diagnoses)*	N	Mean score	SD	p*	N	Mean score	SD	p*	N	Mean score	SD	p*
SCREEN INTERVIEW: PARENT (positive diagnostic areas)												
Any disorder^c^												
Present (1+) Absent	66 12	70.7 64.6	8.1 6.7	.015								
Any affective/anxiety^d^												
Present (1+) Absent					47 31	70.0 62.2	7.9 10.6	< .001				
Any disruptive^e^												
Present (1+) Absent									32 46	72.7 60.9	7.3 7.9	< .001
SCREEN INTERVIEW: CLINICIAN (positive diagnostic areas)												
Any disorder^c^												
Present (1+) Absent	69 9	70.7 62.7	7.9 6.9	.005								
Any affective/anxiety^d^												
Present (1+) Absent					49 29	69.3 62.8	8.1 11.1	.004				
Any disruptive^e^												
Present (1+) Absent									34 44	72.5 60.5	7.0 7.9	< .001
FINAL DIAGNOSES												
Any disorder^c^												
Present (1+) Absent	58 20	71.1 66.1	8.3 6.5	.018								
Any affective/anxiety^d^												
Present (1+) Absent					34 44	69.3 65.1	8.5 10.4	.057				
Any disruptive^e^												
Present (1+) Absent									20 58	74.9 62.5	.2 8.1	< .001

* student T test

^a ^Internalizing problems = Sum of CBCL subscales I, II, III (withdrawal, anxiety/depression, somatic complaints)

^b ^Externalizing problems = Sum of CBCL subscales VII, VIII (delinquent behavior, aggressive behavior)

^c ^One or more diagnostic areas (for the screen interview) or one or more psychiatric disorders (for final diagnoses)

^d ^Any affective disorder (depressive disorders, dysthymia, mania, hypomania, bipolar disorder) and/or any anxiety disorder (social phobia, agoraphobia, specific phobias, separation anxiety disorder, generalized anxiety disorder, obsessive compulsive disorder, panic disorder, acute stress disorder, posttraumatic stress disorder)

^e ^Disruptive disorder (oppositional defiant disorder, conduct disorder)

Regarding K-SADS-PL screen interview, the greater the number of positive diagnostic areas (all 20 areas considered), the higher the CBCL/4-18 total problem scale T-score (parent: r = 0.53, p < .001; clinician: r = 0.55, p < .001). Highly significant correlations (p < .001) were also found between the number of positive affective/anxiety diagnostic areas in the screen interview and CBCL/4-18 internalizing T-scores (parent: r = 0.44; clinician: r = 0.41), and the number of positive disruptive diagnostic areas in the screen interview and CBCL/4-18 externalizing T-scores (parent: r = 0.64; clinician: r = 0.65) (table 4).

**Table 4:** Pearson correlation (r): number of disorders* versus CBCL/4-18 continuous T-scores **

	CBCL/4-18					
	
	Total problems		Internalizing^a^		Externalizing^b^	
			
K-SADS-PL (screen interview and final DSM-IV diagnoses)	r	p	r	p	r	p
SCREEN INTERVIEW: PARENT (positive diagnostic areas)						
Total N of disorders^c^	0.53	< .001				
N of affective/anxiety disorders^d^			0.44	< .001		
N of disruptive disorders^e^					0.64	< .001
SCREEN INTERVIEW: CLINICIAN (positive diagnostic areas)						
Total N of disorders^c^	0.55	< .001				
N of affective/anxiety disorders^d^			0.41	< .001		
N of disruptive disorders^e^					0.65	< .001
FINAL DIAGNOSES						
Total N of disorders^c^	0.50	< .001				
N of affective/anxiety disorders^d^			0.30	.011		
N of disruptive disorders^e^					0.61	< .001

N = Number

* Number of K-SADS-PL disorders: Positive diagnostic areas according to parent or clinician (screen interview), and final DSM-IV diagnoses

** Continuous T-scores for the three CBCL/4-18 broad-band scales: total problems, internalizing problems and externalizing problems

^a ^Internalizing problems = Sum of CBCL subscales I, II, III (withdrawal, anxiety/depression, somatic complaints)

^b ^Externalizing problems = Sum of CBCL subscales VII, VIII (delinquent behavior, aggressive behavior)

^c ^One or more diagnostic areas (for the screen interview) or one or more psychiatric disorders (for final diagnoses)

^d ^Any affective disorder (depressive disorders, dysthymia, mania, hypomania, bipolar disorder) and/or any anxiety disorder (social phobia, agoraphobia, specific phobias, separation anxiety disorder, generalized anxiety disorder, obsessive compulsive disorder, panic disorder, acute stress disorder, posttraumatic stress disorder)

^e ^Disruptive disorder (oppositional defiant disorder, conduct disorder)

Regarding K-SADS-PL final diagnoses, the greater the number of psychiatric disorders (all disorders considered), the higher the CBCL/4-18 total problem scale T-score (r = 0.50, p < .001). In addition, the greater the number of affective/anxiety disorders, the higher the CBCL/4-18 internalizing scale T-score (r = 0.30, p = .011), and the greater the number of disruptive disorders, the higher the CBCL/4-18 externalizing scale T-score (r = 0.61, p < .001) (table 4).

Finally, when using the cut-off T-score > 63 to look at the sensitivity of the three broad-band scales of CBCL/4-18 compared to related K-SADS-PL final diagnoses, 82.8% of children with one or more psychiatric disorders obtained a T-score in the clinical range of the CBCL/4-18 total behavior problem scale, 80.0% of children with any disruptive disorder obtained a T-score in the clinical range of the externalizing scale, and 73.5% of children with any affective/anxiety disorder obtained a T-score in the clinical range of the internalizing scale. When lowering the cut-off (≥ 60) to include borderline children/adolescents in the CBCL/4-18 positive group (with psychopathology), the total behavior problem scale identified 89.7% of children with any psychiatric disorder, the externalizing scale identified 94.3% of children with any disruptive disorder, and the internalizing scale identified 85.3% of children with any affective/anxiety disorder (table 5).

**Table 5:** Sensitivity/specificity of CBCL versus K-SADS-PL considering different cut-off points*

	Sensitivity		Specificity	
		
CBCL/4-18 broad-band scales	T-score > 63^a^	T-score ≥ 60^b^	T-score ≤ 63^c^	T-score < 60^d^
Total problems^e^	82.8	89.7	35.0	20.0
Internalizing^f^	73.5	85.3	36.3	22.7
Externalizing^g^	80.0	94.3	55.9	41.9

* Sensitivity and specificity of CBCL/4-18 broad-band scales compared to related K-SADS-PL final diagnoses according to different CBCL/4-18 T-score cut-off points (N = 78)

^a ^Positive cases on CBCL/4-18 are those with T-scores in the clinical range (> 63)

^b ^Positive cases on CBCL/4-18 are those with T-scores in the clinical/borderline range (≥ 60)

^c ^Negative cases on CBCL/4-18 are those with T-scores in the borderline/normal range (≤ 63)

^d ^Negative cases on CBCL/4-18 are those with T-scores in the normal range (< 60)

^e ^Compared to any psychiatric disorder according to K-SADS-PL final diagnoses

^f ^Compared to any affective/anxiety disorder according to K-SADS-PL final diagnoses

^g ^Compared to any disruptive disorder according to K-SADS-PL final diagnoses

Regarding specificity, when using the cut-off T-score ≤ 63 to identify normal children/adolescents, the CBCL/4-18 identified 35.0% of non-disordered children as borderline or non-clinical in the total problem scale, 55.9% of children with no disruptive disorders as borderline or non-clinical in the externalizing scale, and 36.3% of children with no affective/anxiety disorders as borderline or non-clinical in the internalizing scale. It is important to highlight that non-disordered children according to K-SADS-PL final diagnoses included not only asymptomatic children but also sub-threshold children. In addition, when using the cut-off T-score < 60 to examine the specificity of the three broad-band scales of CBCL/4-18 compared to related K-SADS-PL final diagnoses, 20.0% of non-disordered children were considered non-clinical by the total problem scale, 41.9% of children with no disruptive disorders were considered non-clinical by the externalizing scale, and 22.7% of children with no affective/anxiety disorders were considered non-clinical by the internalizing scale (table 5).

## Discussion

Child mental health research conducted with valid and reliable standardized methods of assessment contributes to data reliability, and increases the possibility of adequate cross-cultural comparisons. Valid diagnostic instruments are fundamental to accurately identify children in need of specialized mental health treatment, and to establish health policies based on the prevalence of mental disorders in different child and adolescent populations. In addition, learning about childhood disorders outside the English-language sphere of influence is very important for establishing service-delivery needs in those regions.

In validity studies involving the use of instruments to evaluate child psychopathology, child psychiatric diagnoses obtained from structured or semi-structured interviews have been compared to behavior checklists' scores based on parental information [19]. Significant relations between CBCL data and results from different diagnostic interviews in child and adolescent psychiatry has long been reported [9,11,20-23], suggesting a substantial convergence between two different approaches used to assess child psychopathology. According to Kasius et al. [24] clinical-diagnostic and empirical-quantitative approaches do not converge to a degree that one approach can replace the other. Despite the important content differences at the item-symptom level between available problem checklists and criteria for psychiatric disorders used by many clinicians and researchers [3], both approaches are needed, useful and complementary.

Although our sample can be considered small, it is compatible with sample sizes of other validity studies regarding psychiatric interview schedules for children and adolescents [25]. In our study, highly significant relations were found between K-SADS-PL and CBCL/4-18 in a relatively small clinical sample of children and adolescents. Because small relations can be proven significant only in large samples [26], our results represent a strong evidence of the convergent validity of K-SADS-PL by comparison with CBCL/4-18.

In addition, the lack of children from the general population in the study sample (to increase the number of non-disordered children) is a study limitation that must be recognized, since study results could have varied as a consequence of sample composition. However, this limitation is minimized by the fact that not only professionals but parents themselves were sources of referral in the current study, resulting in a heterogeneous sample of children with the presence of children without disorders and clinical cases of different severity levels.

As expected, the Brazilian version of K-SADS-PL showed evidence of convergent validity when compared to CBCL/4-18, since both instruments were developed to measure the same construct (child and adolescent psychopathology). Our results showed higher CBCL/4-18 T-scores in children: (1) positive in one or more screen diagnostic areas compared to children negative in all investigated areas; (2) with one or more psychiatric disorders compared to children with no disorders; (3) with greater number of positive screen diagnostic areas; and (4) with greater number of psychiatric disorders. Our validity results were very similar to those reported by the authors of the original K-SADS-PL. In the study of Kaufman et al. [11], CBCL/4-18 internalizing and externalizing scales were used as indices to determine validity of the original K-SADS-PL in a sample of 66 children aged 7-17 years (55 outpatients and 11 controls). In that study, children who screened positive for current depression scored significantly higher on CBCL/4-18 internalizing scale than children who screened negative for current depression (67.5+9.7 vs. 55.6+14.4; p < .0005); and children who screened positive for any current anxiety disorder scored significantly higher on CBCL/4-18 internalizing scale than children who screened negative for any current anxiety disorder (65.2+11.5 vs. 54.4+14.9; p < .003). In addition, children who screened positive for any current behavioral disorder scored significantly higher on CBCL/4-18 externalizing scale than children who screened negative for any current behavioral disorder (61.1+9.9 vs. 51.7+9.2; p < .0001). Higher CBCL/4-18 mean scores were also noted in children who met criteria for current psychiatric disorders compared to those without current disorders (internalizing scores for any depressive disorder: p < .001; internalizing scores for any anxiety disorder: p < .01; externalizing scores for any behavioral disorder: p < .0001).

Kaufman et al. (1997) [11] found a smaller difference between CBCL/4-18 T-score means when comparing internalizing T-score means in children with and without any anxiety disorder than when comparing externalizing T-score means in children with and without any behavioral disorder. In our sample, the only non-significant p value (.057) was noted when comparing internalizing T-score means in children with and without any affective/anxiety disorder. However, it is important to note that in our study, children positive in at least one affective/anxiety screen diagnostic area obtained a significantly higher CBCL/4-18 internalizing T-score mean than children negative in affective/anxiety screen diagnostic areas. Therefore, one may hypothesize that many children who received high scores in the CBCL/4-18 internalizing scale were sub-threshold cases that did not meet DSM-IV criteria for anxiety disorders and were included in the K-SADS-PL non-disordered group, reducing the difference in internalizing T-score means between disordered and non-disordered children.

Only four other validity studies of K-SADS-PL were found in the literature, three of them involving psychiatric clinical samples of children and/or adolescents with no specific health problems [27-29], and one study evaluating the mental health of children and adolescents with traumatic brain injuries or orthopedic injuries [30]. In Israel, Shanee et al. (1997) [27] examined the consensual validity of the Hebrew version of K-SADS-PL in an adolescent inpatient population (n = 57, age = 6-19 years), comparing the instrument final diagnoses to independent consensual DSM-IV diagnoses based on extensive observation and testing of subjects by the inpatient unit team. The authors reported good to excellent validity of diagnoses based on kappa statistics. In Iran, Ghanizadeh et al. (2006) [29] also reported good to excellent consensual validity of all diagnoses except separation anxiety disorder, anorexia, and encopresis when using kappa statistics to compare final diagnoses obtained by the Farsi version of K-SADS-PL with independent DSM-IV diagnoses made by a child and adolescent psychiatrist (n = 109, age = 4-19 years). That sample included 96 psychiatric outpatients and 13 normal controls. In Korea, Kim et al. (2004) [28] used clinical diagnoses based on DSM-IV criteria as a gold standard to examine the consensual validity of K-SADS-PL in a sample of children and adolescents (n = 91, mean age = 8.8 ± 2.1 years). That sample included 80 psychiatric outpatients with a variety of disorders, and 11 controls with no past or current psychiatric disorders. Based on kappa statistics, consensual validity of threshold and sub-threshold diagnoses were good to excellent for ADHD, fair for tic and oppositional defiant disorder, and poor to fair for anxiety and depressive disorders. The authors also examined the convergent validity of K-SADS-PL and CBCL in a sub-sample of 43 children (subjects with CBCL data available). A Korean version of CBCL, standardized in 1990, was applied to identify children with internalizing and externalizing behavior problems. Children considered positive for psychiatric disorders were those with threshold and sub-threshold K-SADS-PL final diagnoses. Besides the small sample size, a significant association (p = .038) was found between K-SADS-PL behavioral disorders (oppositional defiant disorder and/or conduct disorder) and CBCL externalizing behavior problems. No significant association was found between K-SADS-PL anxiety/depressive disorders and CBCL internalizing problems. Finally, in the Netherlands, Wassenberg et al. (2004) [30] evaluated the convergent validity of K-SADS-PL in comparison to CBCL in a sample of children and adolescents with traumatic brain injuries or orthopedic injuries (n = 72, age = 5-14 years). The authors reported excellent convergence between one or more K-SADS-PL final diagnoses and at least one CBCL broad-band scale in the clinical or borderline range (T-score ≥ 60). However, a poor convergence was noted between one or more K-SADS-PL final diagnoses and the CBCL total problem scale, suggesting that the CBCL total problem scale may underestimate psychopathology in this specific population.

A systematic review of the literature assessed the screening efficiency of CBCL in community and clinical samples using published data [31]. A total of 29 studies met the review inclusion criteria, but only a study conducted in Korea [28] applied the K-SADS-PL as a source of comparison diagnosis. According to this systematic review, the estimated sensitivity of the three broad-band CBCL scales were: 0.66 (CI 95%: 0.60 - 0.73) when comparing total problems to any psychiatric disorder; 0.59 (CI 95%: 0.45 - 0.73) when comparing externalizing problems to any disruptive disorders (conduct disorder or oppositional defiant disorder); and 0.61 (CI 95%: 0.47 - 0.75) when comparing internalizing problems to any depression/anxiety disorders. Compared to this systematic review data, our sensitivity results for the CBCL total problem scale, externalizing scale and internalizing scale are higher than the higher limit of the three related 95% confidence intervals, particularly when using the cut-off T-score ≥ 60 (clinical and borderline cases considered positive for psychopathology). The high sensitivity of the three broad-band CBCL scales in our study may be explained by the use of face-to-face interviews to apply the CBCL to parents/caregivers (most of them low-educated), the application of the K-SADS-PL by an experienced child psychiatrist, and the rigorous methodological procedures adopted in our research. In addition, according to this review, the estimated specificity of the three broad-band CBCL scales were: 0.83 (CI 95%: 0.81 - 0.85) when comparing total problems to any psychiatric disorder; 0.79 (CI 95%: 0.65 - 0.94) when comparing externalizing problems to any disruptive disorders; and 0.76 (CI 95%: 0.62 - 0.91) when comparing internalizing problems to any depression/anxiety disorders. In our study, the low specificity of CBCL scales was probably related to the scarcity of asymptomatic children in the studied sample, but because a screening instrument of high sensitivity is extremely useful in identifying children and adolescents in need of further mental health evaluation in the general population, it is worthwhile to maintain the cut-off T-score ≥ 60 to maximize sensitivity at the cost of low specificity. However, further research is needed to find the appropriate CBCL cut-off T-score to identify children and adolescents free of psychopathology in community samples.

## Conclusions

The Brazilian version of K-SADS-PL is a valid instrument to be applied in clinical practice and research involving the mental health of Brazilian children. It showed evidence of convergent validity when compared to CBCL/4-18 in a sample characterized by maternal low education and family low living standards. However, further research needs to address the external validity of the instrument in community-based samples of different regions of Brazil.

K-SADS-PL and CBCL can be used in community samples, school-based samples and clinical samples of school-aged children from all socioeconomic strata. However, to get reliable data from the use of K-SADS-PL, the instrument must be applied by experienced and well-trained clinicians, familiar with DSM-IV criteria. In addition, when the study sample includes low-educated mothers, the CBCL should be applied by a trained interviewer (who may be a lay person). Self-fulfillment must be restricted to samples in which all informants completed at least grade eight.

## Competing interests

The authors declare that they have no competing interests.

## Authors' contributions

Both authors planned the study, participated in data analysis, data interpretation, drafting and critical review of this manuscript, and have read and approved the final manuscript.

## Pre-publication history

The pre-publication history for this paper can be accessed here:

http://www.biomedcentral.com/1471-244X/10/83/prepub
